# Urinary exosomes derived circRNAs as biomarkers for chronic renal fibrosis

**DOI:** 10.1080/07853890.2022.2098374

**Published:** 2022-07-12

**Authors:** Yuhan Cao, Yuanhui Shi, Yanlang Yang, Zhangli Wu, Nana Peng, Jie Xiao, Fan Dou, Jingjing Xu, Wenjun Pei, Cong Fu, Pingsheng Chen, Yuwei Wang

**Affiliations:** a Department of Nephrology, Yi Ji Shan hospital affiliated to Wan Nan Medical College, Wuhu, China; b Key Laboratory of Non-coding RNA Transformation Research of Anhui Higher Education Institution (Wan Nan Medical College), Wuhu, China; c Anesthesia Laboratory & Training Center of Wan Nan Medical College, Wuhu, China; d School of Clinical Medicine, Wan Nan Medical College, Wuhu, China; e School of Anesthesiology, Wan Nan Medical College, Wuhu, China; f Anhui Province Key Laboratory of Biological Macromolecules Research (Wan Nan Medical College), Wuhu, China; g Department of Cardiology, Yi Ji Shan Hospital Affiliated to Wan Nan Medical College, Wuhu, China; h Department of Pathology and Pathophysiology, Medical School, Southeast University, Dhaka, Bangladesh

**Keywords:** CKD, renal fibrosis, urine exosomes, circRNAs, biomarker

## Abstract

**Background:**

Chronic renal disease (CKD) is a common and irreversible loss of renal function. Renal fibrosis reflected the degree of renal dysfunction. However, the current biomarkers only characterize the renal function instead of indicating the fibrosis degree. The potential diagnostic value of urinary exosomes derived circRNAs for renal fibrosis needs to be further studied.

**Methods:**

Urine exosomes from 3 chronic kidney disease (CKD) patients without renal fibrosis and 3 renal fibrotic patients were collected and human circRNAs microarray analysis were performed to detect the circRNAs expression profile. 110 biopsy-proven CKD patients and 54 healthy controls were enrolled and urine exosomes derived RNA was isolated. The expression of hsa_circ_0036649 was measured and the correlation with renal function parameter and pathological indicators was performed. The receiver operating characteristic (ROC) curve for the diagnosis of renal fibrosis was calculated.

**Results:**

Human circRNAs microarray showed 365 circRNAs up expressed and 195 circRNAs down expressed in renal fibrotic patients compared to none fibrosis CKD patients. The expression of hsa_circ_0036649 was decreased in renal fibrotic patients according to RT-PCR and correlated with serum creatinine, blood urea nitrogen (BUN), estimated glomerular filtration rate and cystatin c. Further, the expression of hsa_circ_0036649 was correlated with the score of tubulointerstitial fibrosis (TIF) and the score of glomerular sclerosis. The ROC curve showed that hsa_circ_0036649 may predict renal fibrosis at a cut-off value of 0.597 with a sensitivity of 45.5% and specificity of 87.9%.

**Conclusion:**

Expression of urinary exosomes derived hsa_circ_0036649 associated with the degree of renal fibrosis. Its potential role as a biomarker in CKD remained to be supported by further follow-up studies.

**Key Messages**
- circRNAs profile in urine exosomes in renal fibrosis patients was revealed.
- The expression of urine exosomes derived hsa_circ_0036649 was correlated to renal function and fibrosis degree.
- circRNAs derived from urinary exosomes may become a new research direction for biomarkers of renal fibrosis.

## Introduction

Chronic kidney disease (CKD) was a major public health problem worldwide and in China. A recent cross-sectional survey focussed on Chinese people showed that the morbidity of CKD was 10.8% [1]. Renal fibrosis which was characterised by glomerulosclerosis and tubulointerstitial fibrosis (TIF) was the common pathological finding of end-stage renal disease [2]. The diagnosis of renal fibrosis was a long-term and arduous clinical problem.

Renal biopsy was the golden standard for the diagnosis of chronic fibrosis at present. Renal biopsy was an invasive procedure that may cause severe complications such as bleeding. In clinical practice, it was hard to repeat renal biopsy the dynamic detecting process of renal fibrosis development. So far, there was no non-invasive detection method to accurately diagnose renal fibrosis [3]. In recent years, urine biomarkers attracted researchers. Li et al. firstly established a quantitative PCR method for urinary RNA detection [4]. The development of microarray technology made urine RNA detection become a research hotspot. Recently, the role of non-coding RNA such as circRNAs in CKD had become one of the hot issues [5]. CircRNAs had the potential to become a biomarker of kidney disease [6,7].

Exosomes were a kind of micro-lipid double-layer microcapsule structure formed by the fusion of multi-capsular complex and cell membrane with a diameter of 40–100nm. Exosomes captured some biomolecules, including proteins and RNA, from the cytoplasm of the cells during the formation of exosomes [8,9]. Pisitkun et al. firstly found exosomes in urine [10]. However, if the circRNAs in urine-derived exosomes reflected the CKD and indicated renal fibrosis remained unknown. Accordingly, this study was designed to find the expression of circRNAs in urine exosomes and validated the potential of urine exosomes act as biomarkers for renal fibrosis.

## Methods

### Study population

Firstly, 3 CKD patients without renal fibrosis and 3 renal fibrosis patients were enrolled. The urine samples were collected and the whole RNA in urine exosomes was isolated to be analysed by human circRNAs microarray. Secondly, a total of 110 biopsy-proven CKD patients were selected from the Department of Nephrology, Yi Ji Shan Hospital, Wan Nan medical college. The pathological types of 110 CKD patients were as follows: IgA nephropathy (*n* = 42), membranous nephropathy (*n* = 28), minimal change disease (*n* = 7), focal segmental glomerulosclerosis (*n* = 7), diabetic nephropathy (*n* = 2), hypertensive nephropathy (*n* = 4), intracapillary proliferative glomerulonephritis (*n* = 1), mesangial proliferative glomerulonephritis (*n* = 5), membranoproliferative glomerulonephritis (*n* = 3), crescentic glomerulonephritis (*n* = 1) and minor glomerular abnormalities (*n* = 10). All the CKD patients were divided into none fibrosis group, mild-moderate fibrosis group and severe fibrosis group according to the score of tubulointerstitial fibrosis (TIF). The exclusion criteria were: patients younger than 18 years old or older than 80 years old; patients with the following disorders: chronic liver disease; lung disease; urinary tract infection; cancer; organ transplantation; cardiovascular disorder; Parkinson’s disease; use of steroids or immunosuppressive medications. The results of the laboratory examination and pathological parameters were collected. 54 age- and gender-matched healthy volunteers also enrolled who were defined as the absence of abnormalities on a routine urinalysis and normal renal function [estimated glomerular filtration rate (eGFR)>90 ml^−1^·min^−1^·1.73 *m*^−2^]. The whole stream of early morning urine specimens of age- and gender-matched healthy volunteers were collected.

### Collection of urine samples and exosomes

The whole stream of early morning urine specimens was collected after hospitalisation. Samples were centrifuged at 3,000 *g* for 30 min at 4 °C. Then the supernatants were centrifuged at 13,500 *g* for 30 min at 4 °C. The sediments were discarded and the supernatants were centrifuged at 100,000 *g* for 70 min at 4 °C. The sediments were suspended in 100 μL phosphate buffer saline (PBS). The suspension was urinary exosomes (U-EXO) and was identified by transmission electron microscope (TEM, Hitachi, HT-7700), nanoparticle tracking analysis (NTA) (Particle Metrix’ ZetaView), and western blot (Detection of CD9 and TSG101).

### RNA isolation

The whole RNA in exosomes was isolated using Trizol-LS according to the manufacturer’s protocol. The quality control of RNA was performed using Agilent 2100 Bioanalyzer. The concentration and purity of RNA were assessed using the relative absorbance ratio at 260/280 in a NanoDrop 2000 (Thermo, USA).

### Detection of CircRNAs expression

The circRNAs expression of 3 none fibrosis CKD patients’ U-EXO (NFU-EXO) and 3 renal fibrosis U-EXO (FU-EXO) were tested using LC Human ceRNA Microarray (LianChuan Biotechnology Limited).

### Real-time RT-qPCR

The reverse transcription was performed using PrimeScript^TM^ RT Reagent Kit (TAKARA, Japan) according to the manufacturer’s protocol. RT-PCR was performed using TB Green Premix Ex Taq Kit (TAKARA, Japan). The primers was as follows: hsa_circ_0036649 primers (sense: 5′-TCTCCATTGACAAAATCCATCT-3′; antisense: 5′-GCCTTCTAGACTGAAATGTCCA-3′) and U6 primers (sense: 5′-GCTTCGGCAGCACATATACTAAAAT-3′; antisense: 5′-CGCTTCACGAATTTGCGTGTCAT-3′). Hsa_circ_0036649 expression was normalised to U6 and calculated as 2^−ΔΔCt^.

### Renal fibrosis

The degree of renal fibrosis for CKD patients was evaluated and the Score of TIF and glomerular sclerosis were calculated according to the previous study described [11]. Two experienced pathologists who were blinded to the studies subjectively scored the severity of renal fibrosis. None fibrosis was considered to be 0 of the renal interstitium fibrosis. Mild-moderate fibrosis was considered to be ≤50%. Severe was considered to be >50%.

### Western blot

The protein in U-EXO was collected using RIPA lysing buffer (Beyotime, China). Protein concentration was determined using a BCA kit (Beyotime, China). The CD9 and TSG101 protein expression in U-EXO were determined by western blot. Rabbit anti-human CD9 antibody (PROTEINTECH, USA) and rabbit anti-human TSG101 antibody (PROTEINTECH, USA) were used. HRP-labeled Goat Anti-rabbit IgG (Beyotime, China) was used as the secondary antibody.

### Statistical analysis

SPSS 17.0 was used for data analysis. The method of calculating the relative expression of hsa_circ_0036649 was described in a previous study [11]. Statistical comparison of different types of data, calculate the correlation coefficient, logistic regression and receiver operating characteristic (ROC) curves were calculated according to the previous study described [11]. The *P* value of the deviation of linearity was calculated to confirm the linear relationship between expression of hsa_circ_0003649 and scr, BUN, cycstatin C, eGFR, 24 h proteinuria, a score of TIF and score of glomerular sclerosis (*p* > .05 indicated the linear relationship). All *P* values were two-tailed, and *p* < .05 was considered statistically significant.

## Results

### Exosomes identification

**Figure 1.**
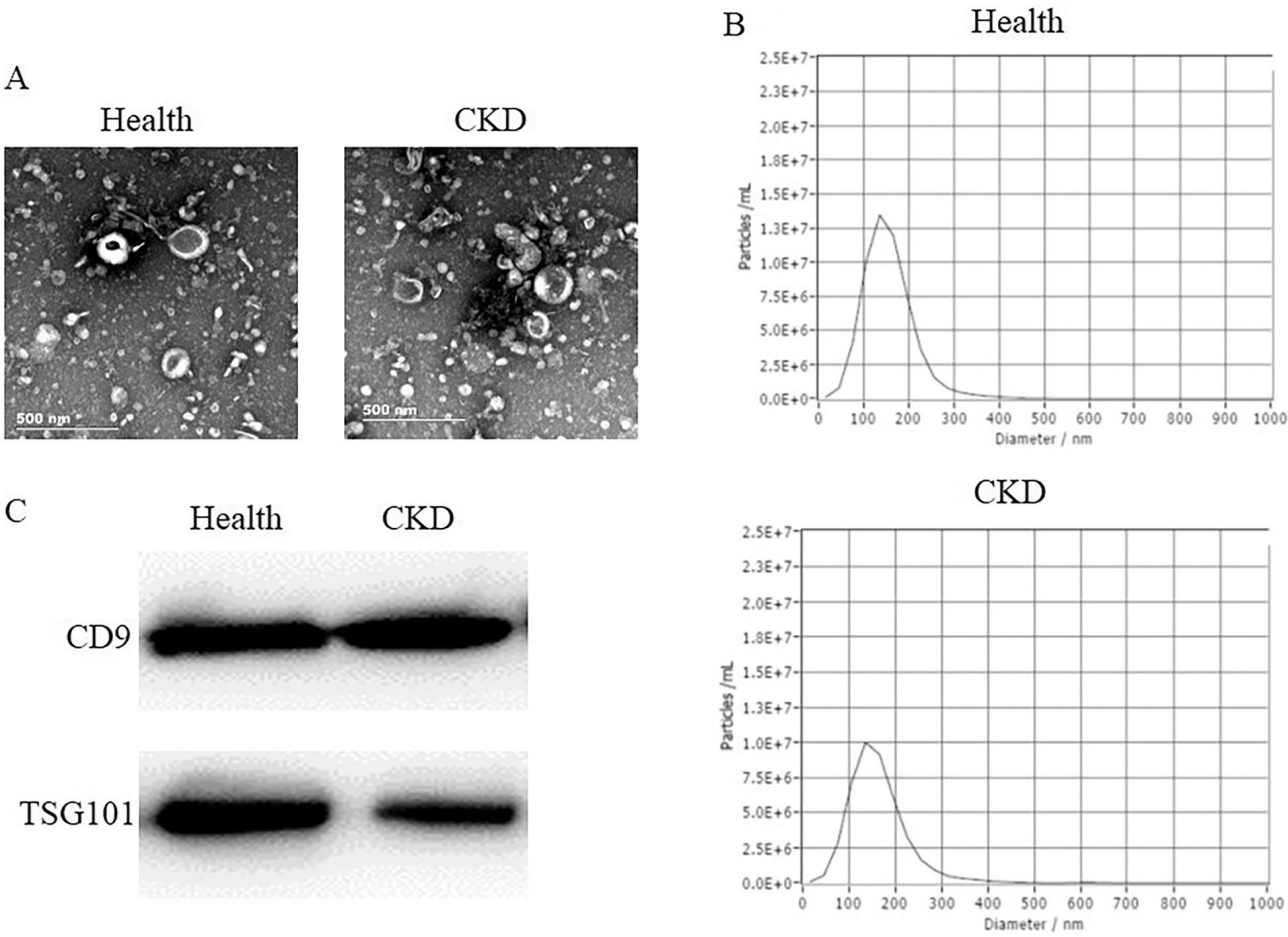
Urinary exosomes identification. (A) TEM image of exosomes from healthy controls and CKD patients. (B) NTA analysis of urinary exosomes. (C) Western blot showed that exosomes from healthy controls and CKD patients expressed CD9 and TSG101.

The TEM image U-EXO were shown in Figure 1A. The NTA shown in Figure 1B indicated the purity of exosomes. The western blot showed that both U-EXO from healthy controls and CKD patients expressed exosome marker CD9 and TSG101 (Figure 1C). Figure S1 showed that the RNA from healthy controls U-EXO and CKD U-EXO had less contamination of cellular RNA.

### CircRNAs expression in U-EXO

**Figure 2.**
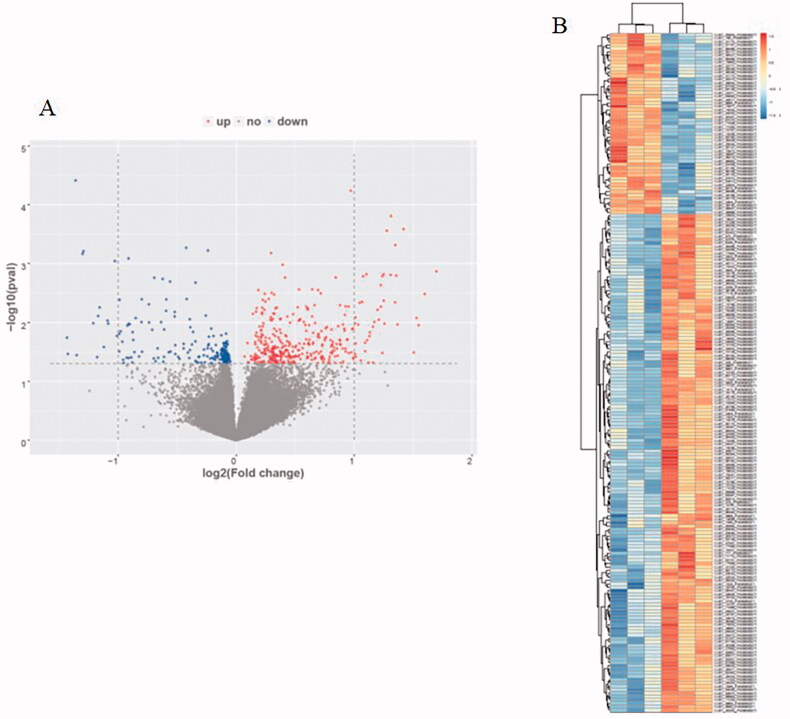
Human circRNAs microarray. (A) Volcano map of circRNAs microarray. (B) Heat map of circRNAs microarray.

We found that 560 circRNAs have a statistical difference in FU-EXO compared to NFU-EXO. Among the differently expressed circRNAs, 365 circRNAs up expressed and 195 circRNAs down expressed in FU-EXO compared to NFU-EXO. Hsa_circ_0036649 which was down expressed in FU-EXO was chosen to be validated using RT-PCR. Figure 2A showed the volcano map and Figure 2B showed the heat map of circRNAs microarray. The clinical information of 3 none fibrosis CKD patients and 3 renal fibrosis patients were shown in Table S1. The Masson staining of 3 renal fibrosis patients and 3 none fibrosis patients were shown in Figure S2.

**Table 1.** Clinical profile of patients with CKD and healthy controls.

	CKD (*n* = 110)	Control (*n* = 54)	*P* value
Age (years)	43.8 ± 15.6	39.2 ± 15.3	.075
Gender (male/female)	62/48	38/16	.084
24h Proteinuria (g/day)	2.315(0.010–24.580)	/	/
Scr (mmol/l)	108.9 ± 83.6	62.1 ± 6.8	<.001
BUN (mmol/L)	7.1 ± 3.8	4.6 ± 1.2	<.001
Cystatin C (mg/L)	1.44 ± 0.88	0.67 ± 0.10	<.001
eGFR (ml/min per 1.73 m^2^)	82.8 ± 35.2	121.7 ± 21.7	<.001
SBP (mmHg)	131.2 ± 15.7	120.3 ± 7.3	<.001
DBP (mmHg)	85.8 ± 12.8	73.7 ± 6.5	<.001
Score of TIF	30 (0–91)	/	
Score of glomerular sclerosis	1.0 (0.0–4.0)	/	/
hsa_circ_0036649	0.642	0.951	/
	(0.187–3.848)	(0.889–0.977)	<.001

Scr: serum creatinine; eGFR: estimated glomerular filtration rate; BUN: blood urea nitrogen; SBP: systolic blood pressure; DBP: diastolic blood pressure.

### Characteristics of patients

**Figure 3.**
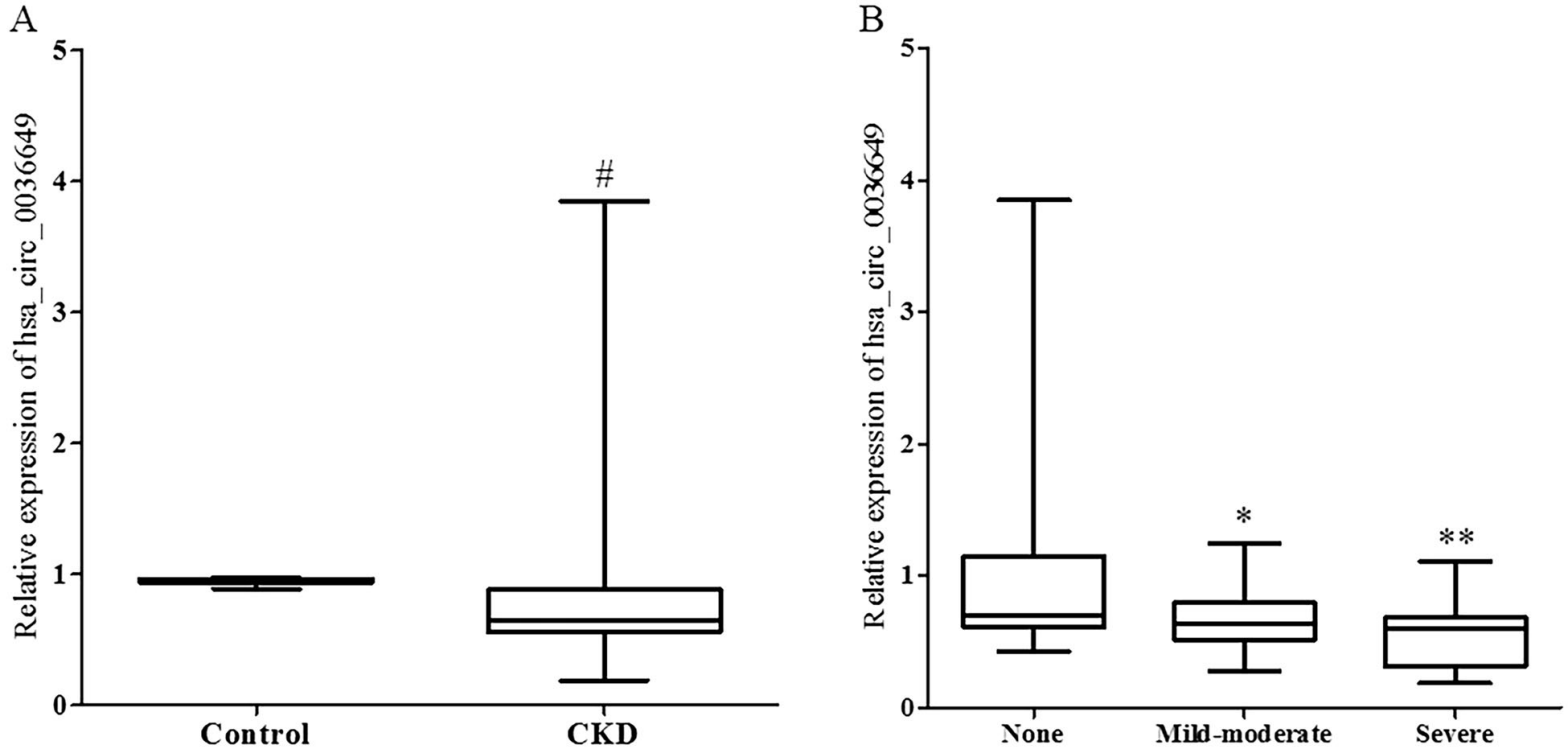
Expression of hsa_circ_0036649 in urine exosomes from CKD patients and healthy control. (A) Expression of hsa_circ_0036649 was significantly decreased in CKD patients compared to healthy controls. (B) In CKD patients, urinary exosomes hsa_circ_0036649 expression was significantly decreased in mild-moderate and severe renal fibrosis patients compared to none fibrosis (#*p* < .001 vs control; *p = .012 vs none fibrosis; **p = .041 vs mild-moderate, *p* < .001 vs none fibrosis).

The primary clinical and pathological characteristics of the involved subjects are shown in Table 1. There were no significant differences in age and gender between CKD patients and controls. The CKD group had a significant increase in Scr, BUN, cystatin c and a decrease in the estimated glomerular filtration rate (eGFR) compared to controls. eGFR was calculated using modified MDRD [12] equations for patients. Relative expression of hsa_circ_0036649 was significantly decreased in the CKD group (Median expression 0.642 in CKD group vs 0.951 in healthy controls, *p* < .001 vs control, Figure 3A).

The 110 CKD patients were divided into 3 groups according to renal fibrosis degree that were evaluated by two experienced pathologists who were blinded to the studies. There was no significant difference between the two pathologists. As shown in Table 2, there were no significant differences in age, gender and 24 h proteinuria among the 3 groups. The relative expression of U-EXO hsa_circ_0036649 was significantly lower in the mild-moderate group and severe group (Median expression 0.635 in mild-moderate vs 0.703 in none fibrosis group, *p* = .012; mild-moderate vs 0.602 in severe, *p* = .041; Figure 3B).

**Table 2.** Clinical and pathological parameters of CKD patients.

	None	Mild-Moderate	Severe	*P* value
	(*n* = 33)	(*n* = 42)	(*n* = 35)	
Age, y	42.0 ± 18.9	41.7 ± 13.4	48.4 ± 15.0	.153
Sex (M/F)	16/17	23/19	23/12	.346
Scr, μmol/L	85.8 ± 67.6	111.2 ± 38.3	134.6 ± 73.5	.036
BUN, mmol/L	5.6 ± 2.8	7.4 ± 4.3	8.6 ± 3.6	.001
Cystatin C, mg/L	1.08 ± 0.60	1.39 ± 0.99	1.92 ± 0.86	<.001
eGFR (ml/min per 1.73 m^2^)	99.9 ± 30.1	88.5 ± 38.3	57.2 ± 20.8	<.001
24h proteinuria (g/day)	1.800	1.595	2.850	.093
	(0.010–9.160)	(0.100–21.580)	(0.220–13.210)	
SBP, mmHg	130.1 ± 15.7	126.6 ± 13.3	137.9 ± 16.7	.006
DBP, mmHg	84.2 ± 12.3	82.1 ± 10.6	91.9 ± 13.7	.002
Urine pH	6.2 ± 0.7	6.2 ± 0.6	6.0 ± 0.6	.324
Usage of diuretic	12	16	13	.988
Usage of ACEI/ARB	17	22	16	.826
hsa_circ_0036649	0.703	0.635	0.602	.001
	(0.429–3.848)	(0.281–1.250)	(0.187–1.111)	
Score of TIF	0	30 (0–45)	70 (52–91)	<.001
Score of glomerular sclerosis	0.6 (0.0–0.9)	1.0 (0.6–2.4)	2.2 (0.9–4.0)	<.001

Scr: serum creatinine; eGFR: estimated glomerular filtration rate; BUN: blood urea nitrogen; SBP: systolic blood pressure; DBP: diastolic blood pressure.

### Correlation between U-EXO hsa_circ_0036649, clinical and pathological parameters

**Figure 4.**
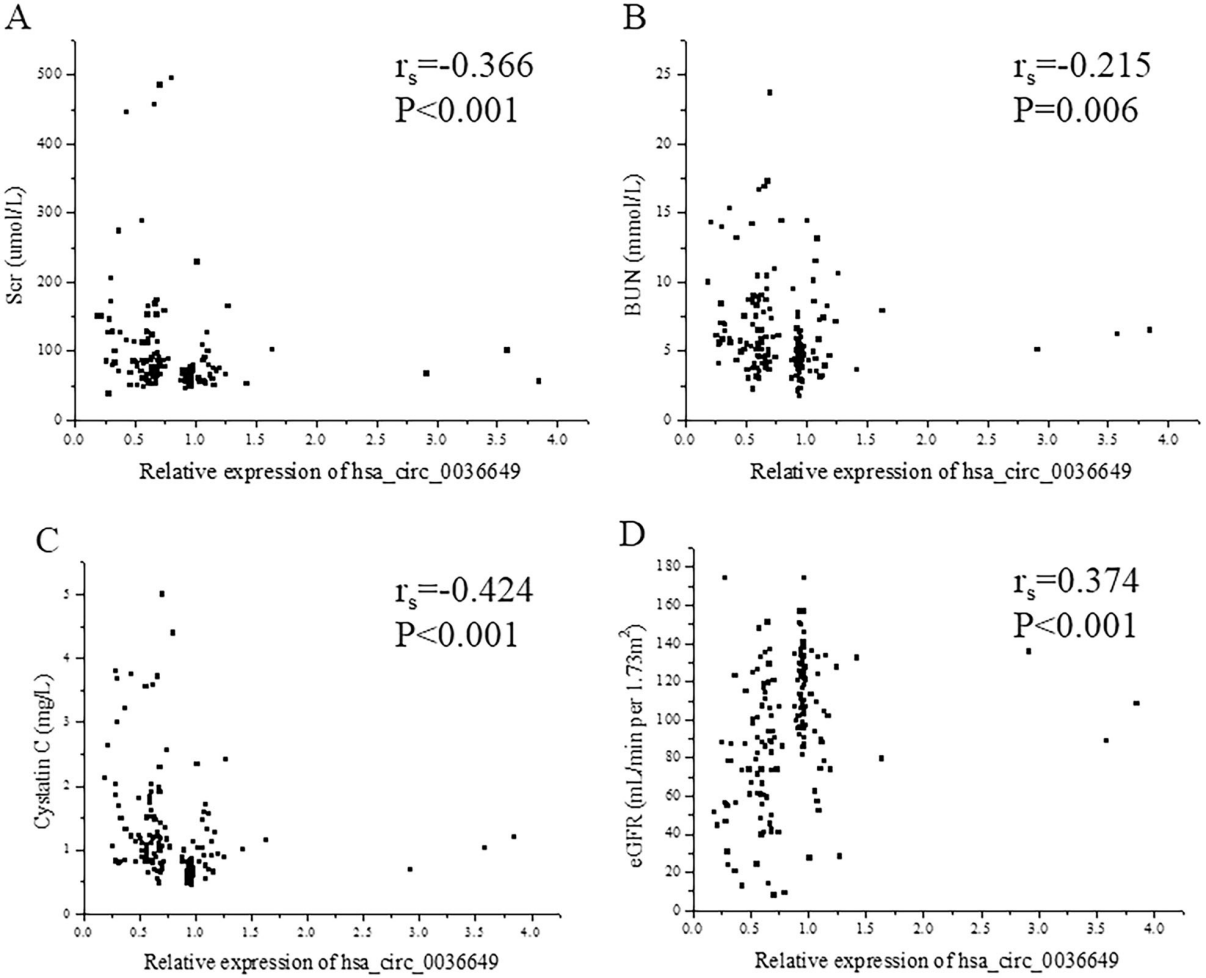
Correlation between hsa_circ_0036649 expression and clinical parameters. (A) Spearman correlation between hsa_circ_0036649 and Scr (r_s_ = −0.366, *p* < .001). (B) Spearman correlation between hsa_circ_0036649 and BUN (r_s_ = −0.215, *p* = .006). (C) Spearman correlation between hsa_circ_0036649 and cystatin c (r_s_ = −0.424, *p* < .001). (D) Spearman correlation between hsa_circ_0036649 and eGFR (r_s_ = 0.374, *p* < .001).

**Figure 5.**
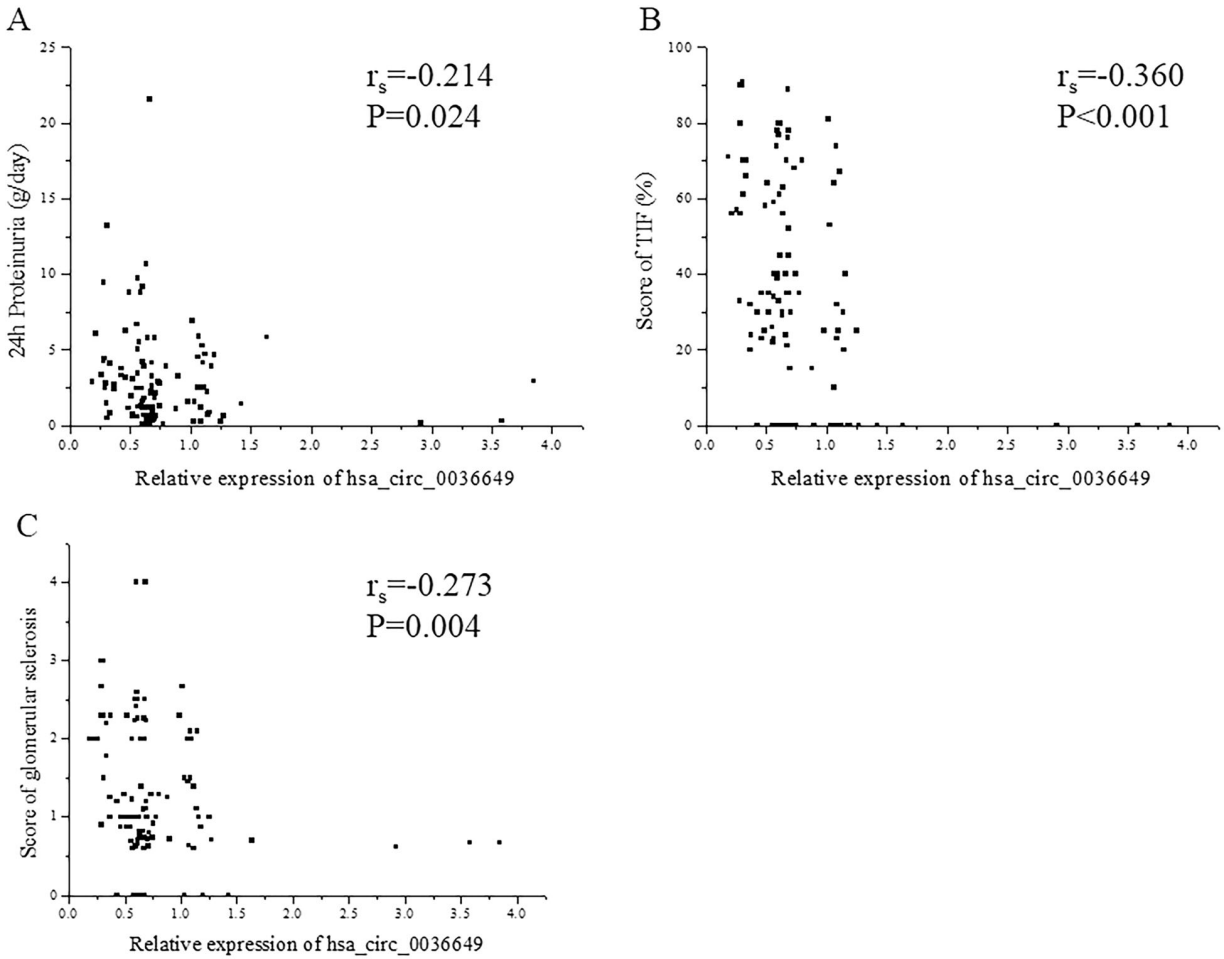
Correlation between hsa_circ_0036649 expression and 24 h Proteinuria, renal fibrosis pathological parameters. (A) Spearman correlation between hsa_circ_0036649 and 24 h Proteinuria (r_s_ = −0.214, *p* = .024). (B) Spearman correlation between hsa_circ_0036649 and score of TIF (r_s_ = −0.360, *p* < .001). (C) Spearman correlation between hsa_circ_0036649 and score of glomerular sclerosis (r_s_ = −0.273, *p* = .004).

The P value of deviation of linearity between expression of hsa_circ_0003649 and scr, BUN, cycstatin C and eGFR was 0.103, 0.084, 0.145 and 0.157, respectively. U-EXO hsa_circ_0036649 correlated with Scr (r_s_ = −0.366, *p* < .001; Correlation equation: *Y* = 118.11–29.83X, *p* = .018), BUN (r_s_ = −0.215, *p* = .006; Correlation equation: *Y* = 7.08–0.99X, *p* = .029), cystatin c (r_s_ = −0.424, *p* < .001; Correlation equation: *Y* = 1.56–0.45X, *p* = .001) and eGFR (r_s_=0.374, *p* < .001; Correlation equation: *Y* = 78.42 + 20.90X, *p* < .001; Figure 4). In CKD patients, The P value of deviation of linearity between expression of hsa_circ_0003649 and 24 h proteinuria, score of TIF and score of glomerular sclerosis was 0.087, 0.360 and 0.081, respectively. U-EXO hsa_circ_0036649 correlated with 24 h proteinuria (r_s_ = −0.214, *p* = .024; Correlation equation: *Y* = 3.62–0.83X, *p* = .014), score of TIF (r_s_ = −0.360, *p* < .001; Correlation equation: *Y* = 47.75–18.54X, *p* < .001) and score of glomerular sclerosis (r_s_ = −0.273, *p* = .004; Correlation equation: *Y* = 1.61–0.38X, *p* = .019; Figure 5).

Stepwise multivariate logistic regression analysis further showed that the relative expression of U-EXO hsa_circ_0036649 significantly correlated with renal fibrosis (Table 3, OR, 0.125; 95% CI, 0.026–0.599; *p* = .009). Scr, BUN, cystatin c, eGFR and 24 h proteinuria had no statistical significance in stepwise multivariate logistic regression analysis (Table 3).

**Table 3.** Multivariate logistic regression analysis of selected variables for renal fibrosis.

	OR	95% CI	*P* Value
Hsa_circ_0036649	0.125	0.026–0.599	.009
Scr	0.995	0.983–1.007	.383
BUN	0.888	0.714–1.105	.287
Cystatin C	1.465	0.319–6.737	.623
eGFR	0.986	0.965–1.008	.217
24h proteinuria	1.080	0.904–1.289	.398

Scr: serum creatinine; eGFR: estimated glomerular filtration rate; BUN: blood urea nitrogen; OR: odds ratio; CI: confidence interval.

### Diagnostic value of U-EXO hsa_circ_0036649 for renal fibrosis

**Figure 6.**
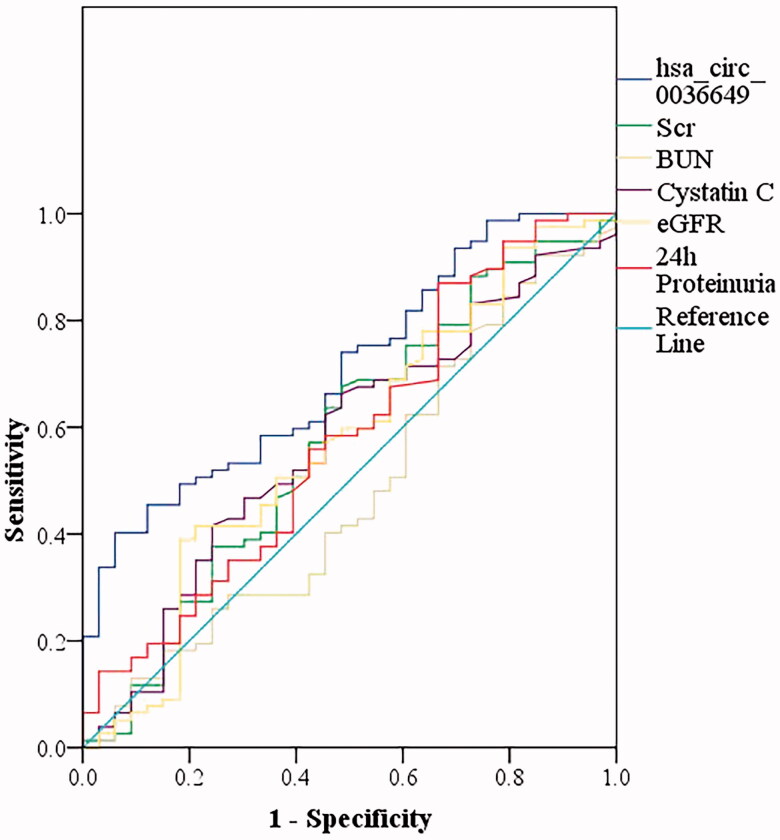
The receiver operating characteristic (ROC) curve showed the diagnosis value of the urine exosomes hsa_circ_0036649 for renal fibrosis. ROC curve showed the urine exosomes hsa_circ_0036649 distinguished renal fibrosis (AUC of 0.706, 95% CI, 0.606–0.807; *p* = .001).

The results showed that U-EXO hsa_circ_0036649 effectively distinguished renal fibrosis, with the largest AUC of 0.706 (95% CI, 0.606–0.807; *p* = .001) higher than that of Scr (AUC of 0.575; 95% CI, 0.453−0.697; *p* = .214), BUN (AUC of 0.479; 95% CI, 0.358–0.601; *p* = .732), cystatin c (AUC of 0.572; 95% CI, 0.454–0.689; *p* = .235), eGFR (AUC of 0.573; 95% CI, 0.452–0.695; *p* = .224) and 24 h proteinuria (AUC of 0.579; 95% CI, 0.459–0.700; *p* = .189). U-EXO hsa_circ_0036649 displayed the sensitivity of 45.5% and specificity of 87.9% at the optimal cut-off value of 0.597 (Figure 6).

## Discussion

Our study firstly indicated the different expression of circRNAs in urine exosomes between none renal fibrosis patients and renal fibrosis patients. Furthermore, urinary exosomes derived circRNAs had the potential to become the non-invasive biomarkers for renal fibrosis.

Early, accurate and non-invasive diagnosis of renal fibrosis was important in treating CKD. Accurate biomarkers can not only replace traumatic pathological examination but also guided treatment and prompt prognosis. Some studies had found that markers of key biological events in renal fibrosis can be detected before pathological changes appear, and were related to the risk of progression to fibrosis [13]. For a long time, urine had become the main source of non-invasive biomarkers for the diagnosis of kidney disease because it carried a lot of kidney disease-related information. Up to now, it had been found that there were many small cells in the urine, including podocytes and tubular epithelial cells. In 2001, Li et al. firstly established a non-invasive approach to diagnosing acute renal rejection of allografts by isolating and quantifying RNA of specific genes in urine cells [4]. Urinary sediment had attracted researchers' focus and had become an attractive resource for detecting the biomarker for kidney diseases [11,14–17]. However, the current urine cell mRNA detection technology still had some limitations. There were a large proportion of non-renal cells in the urine, such as transitional epithelial cells and squamous epithelial cells. Kidney proper cells such as renal tubular epithelial cells (TEC) and podocytes were mixed in different proportions [18]. mRNA expression level in urine cannot reflect the degree of renal disease accurately [19].

Exosomes captured some of the biomolecules from the cytoplasm of the mother cells during their formation, including protein and RNA. Pisitkun et al. firstly discovered exosomes in urine in 2004 [10]. Urine exosomes were produced by the fusion of MVB with renal epithelial cells (such as glomerular podocytes, renal tubular epithelial cells and epithelial cells of other urinary pathways) which can reflect the pathological changes in the kidney [20]. Nucleic acid information from different parts of the nephron was included in urine exosomes [21]. Exosomes had been proposed to play roles in cell-cell communications within nephron segments that affected renal physiology [22]. Urine exosomes derived protein [23,24], mRNA [25] and miRNA [26,27] had been found the diagnostic value for glomerular diseases. In our study, we isolated urinary exosomes from none fibrosis CKD patients and renal fibrosis patients using ultracentrifugation and detected the circRNAs expression profile. Furthermore, we found the circRNAs expression difference between none fibrosis and fibrosis and determined the diagnostic potential of urine exosome circRNAs. CircRNAs was an important candidate for non-invasive biomarkers of renal fibrosis.

CircRNAs were a class of non-coding linear RNA molecules. Because circRNAs were not sensitive to nuclease, it was more stable than linear RNA. CircRNAs were very suitable for clinical detection as a non-invasive biomarker because of the stable cyclic structure that hardly to is degraded by RNases in body fluids. A previous study showed that exosomes contained abundant circRNAs [28]. Exosomal circRNAs had potential applications as disease biomarkers [29]. So far, the study of urinary exosome circular RNA and kidney disease was very limited. Ma et al. reported that 89 circRNAs were significantly differentially expressed in idiopathic membranous nephropathy patients’ urine exosomes and MUC3A could be considered a potential diagnostic biomarker of idiopathic membranous nephropathy [7]. In this study, we found that 560 circRNAs were significantly differentially expressed in renal fibrosis patients’ urinary exosomes using a human circRNAs microarray. We further found that hsa_circ_0036649 was down expressed in renal fibrosis patients. Urinary exosomal hsa_circ_0036649 had good diagnostic efficacy for renal fibrosis and was better than creatinine. Creatinine was the gold standard to reflect renal function. A previous study found that the correlation between urinary mRNA and renal fibrosis was better than creatinine [14]. Furthermore, Cao et al. found that urinary mRNA was better than creatinine in the diagnosis of renal fibrosis [11]. In clinical practice, it was often observed that creatinine was only slightly elevated in patients with moderate and severe renal fibrosis. Due to the strong compensatory capacity of the kidney, creatinine may not exceed the normal range in moderate renal fibrosis. With the continuous development of renal fibrosis, more and more cells lost function, fewer and fewer healthy nephrons, and creatinine gradually increased. However, the current biomarkers such as scr, BUN and cycstatin C only indicated the renal function instead of indicating the fibrosis degree. Our study suggested that biomarkers in urine may have better diagnostic efficacy for renal fibrosis than creatinine. Hsa_circ_0036649 well correlated with renal function parameters and renal pathological score and had the potential to be acted as non-invasive biomarkers for renal fibrosis. Urinary exosomal circRNAs played an important role in the early non-invasive diagnosis of renal fibrosis according to our data. However, due to the small sample of this study, urinary exosomal circRNAs testing cannot replace renal biopsy in clinical practice. circRNAs derived from urinary exosomes may become a new research direction for biomarkers of renal fibrosis.

Hsa_circ_0036649 originated from chr15:86207793-86213061 and the gene symbol was AKAP13. AKAP13 was a Rho guanine nucleotide exchange factor regulating activation of RhoA, which was known to be involved in profibrotic signalling pathways. At present, fewer studies indicated the role of hsa_circ_0036649 in renal fibrosis. A previous study revealed that AKAP13 was expressed in the alveolar epithelium and lymphoid follicles of patients with idiopathic pulmonary fibrosis, and AKAP13 mRNA expression was higher in lung tissue of patients with IPF than that in lung tissue from controls [30]. Other studies indicated that circRNA_010383 [31], circACTR2 [32], circ_0000064 [33] and circRNA_30032 [34] played a key role in renal fibrosis in mice model or cell lines. In this study, we found that hsa_circ_0036649 had a diagnostic value for renal fibrosis. Urine exosomes derived circRNAs were an important resource for finding non-invasive biomarkers for renal fibrosis. However, if hsa_circ_0036649 regulated renal fibrosis and the detailed mechanism needed to be further studied.

Our study also had some limitations. Firstly, the involved CKD patients contained several pathological types. It was unclear if urine exosomes derived circRNA can diagnosis renal fibrosis in a specific pathological type. Secondly, the sample size of this study was still small, which needs to be further verified by a large-scale study. We do not perform a follow-up study to determine the relationship between urine exosomes derived hsa_circ_0036649 and the progression of renal fibrosis. Additionally, cell and animal experiments were needed to verify the regulatory effect of hsa_circ_0036649 on renal fibrosis and the mechanism of urinary exosomal circRNAs regulating renal fibrosis needed to be further studied.

## Conclusion

Expression of urinary exosomes derived hsa_circ_0036649 associated with the degree of renal fibrosis. Its potential role as a biomarker in CKD remained to be supported by further follow-up studies. Urinary exosomal circRNAs may become a new research direction for biomarkers of renal fibrosis.

## Supplementary Material

Supplemental Material

## Data Availability

The datasets used and/or analysed during the current study are available from the corresponding author on reasonable request.
