# Supplementary material for: Urinary exosomes derived circRNAs as biomarkers for chronic renal fibrosis
Source: Ann Med. 2022 Jul 12;54(1):1966–76. doi: 10.1080/07853890.2022.2098374 (PMC9291679; doi:10.1080/07853890.2022.2098374)
Supplement: Supplemental Material [file IANN_A_2098374_SM0433.zip › Supplemental files/Table S1.docx]

**Table S1. Characteristics of patients involved in human circRNAs microarray analysis.**

|  |  | None fibrosis (n=3) | | |  |  |  | Fibrosis  (n=3) |  | p |
| --- | --- | --- | --- | --- | --- | --- | --- | --- | --- | --- |
|  | Patient 1 | Patient  2 | | Patient 3 | |  | Patient 1 | Patient 2 | Patient 3 |  |
| 24h Proteinuria (g/day) | 3.054 | 3.318 | 0.28 | | |  | 0.5 | 0.65 | 2.87 | 0.517 |
| Scr (mmol/L) | 61 | 263 | 48.8 | | |  | 679.4 | 42.1 | 150.3 | 0.470 |
| BUN (mmol/L) | 5.2 | 26.4 | 8.74 | | |  | 23.66 | 4.27 | 9.98 | 0.930 |
| Cystatin C (mg/L) | 1.26 | 2.05 | 1.31 | | |  | 5.48 | 0.79 | 2.13 | 0.424 |
| eGFR(ml/min per 1.73m^2^) | 109.5 | 24.74 | 125.4 | | |  | 10.9 | 169.0 | 43.4 | 0.843 |
| SBP (mmHg) | 130 | 125 | 130 | | |  | 157 | 112 | 139 | 0.592 |
| DBP (mmHg) | 64 | 75 | 80 | | |  | 109 | 80 | 95 | 0.087 |
| Pathological type | minor glomerular abnormalities | minor glomerular abnormalities | Membranous nephropathy | | |  | focal segmental glomerulosclerosis | IgA nephropathy | membranoproliferative glomerulonephritis |  |
| Score of TIF (%) | 0 | 0 | 0 | | |  | 75 | 25 | 55 | 0.024 |
| Score of glomerular sclerosis | 0 | 1 | 0 | | |  | 2.5 | 1 | 0.74 | 0.168 |
