# Supplementary figures and images for: Urinary exosomes derived circRNAs as biomarkers for chronic renal fibrosis
Source: Ann Med. 2022 Jul 12;54(1):1966–76. doi: 10.1080/07853890.2022.2098374 (PMC9291679; doi:10.1080/07853890.2022.2098374)

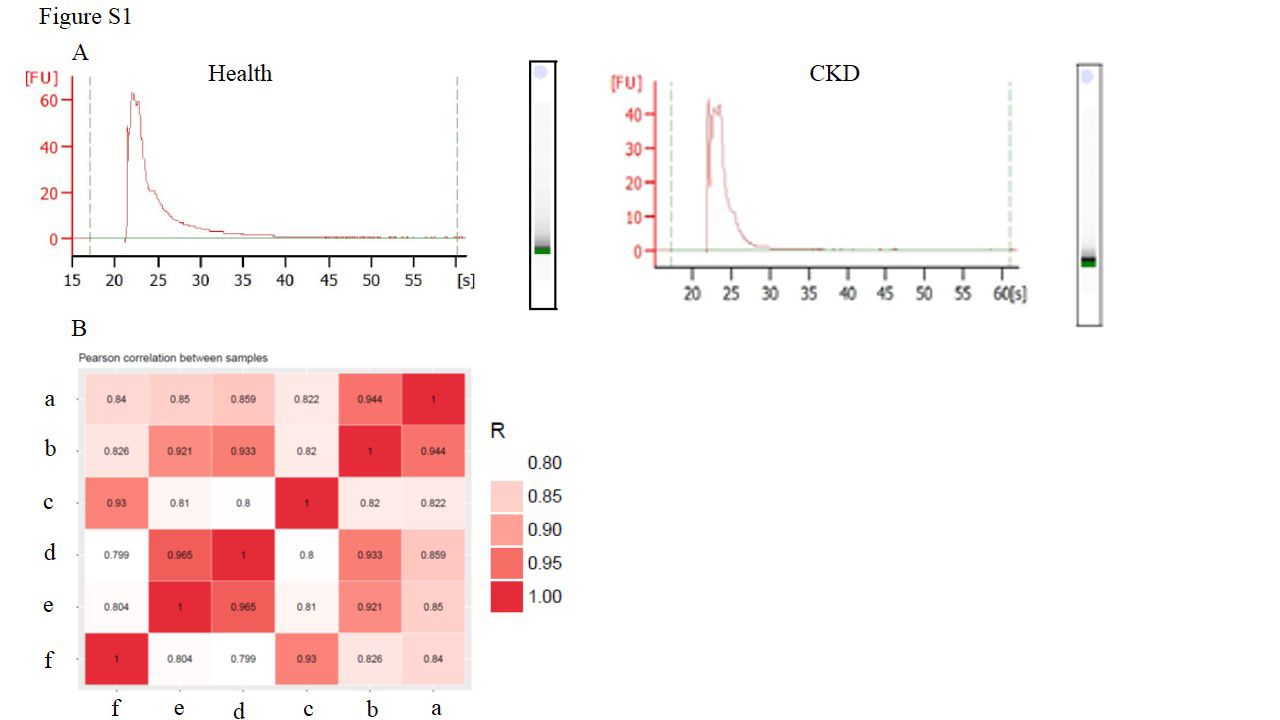

Supplement: Supplemental Material [file IANN_A_2098374_SM0433.zip › Supplemental files/figure S1.jpg]

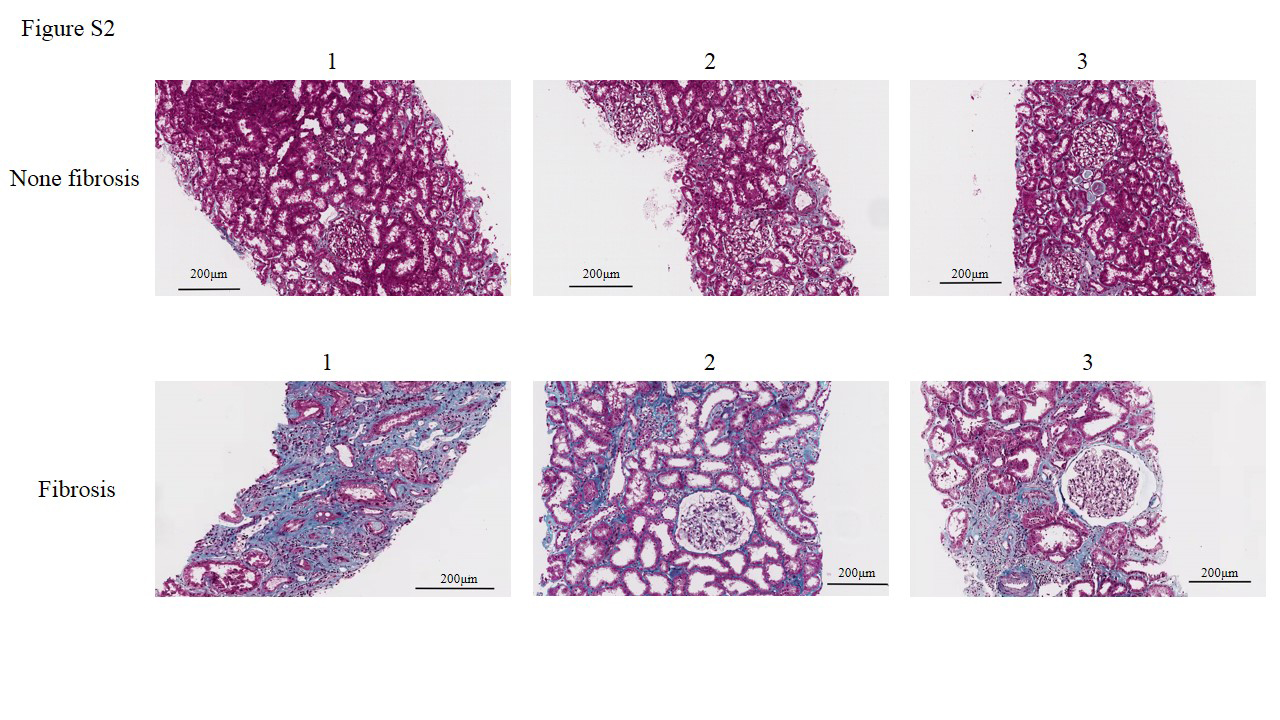

Supplement: Supplemental Material [file IANN_A_2098374_SM0433.zip › Supplemental files/figure S2.jpg]
